# T-Cell Assays for Tuberculosis Infection: Deriving Cut-Offs for Conversions Using Reproducibility Data

**DOI:** 10.1371/journal.pone.0001850

**Published:** 2008-03-26

**Authors:** Anandharaman Veerapathran, Rajnish Joshi, Kalyan Goswami, Sandeep Dogra, Erica E. M. Moodie, M. V. R. Reddy, Shriprakash Kalantri, Kevin Schwartzman, Marcel A. Behr, Dick Menzies, Madhukar Pai

**Affiliations:** 1 Department of Biochemistry, Mahatma Gandhi Institute of Medical Sciences, Sevagram, Maharashtra, India; 2 Department of Medicine, Mahatma Gandhi Institute of Medical Sciences, Sevagram, Maharashtra, India; 3 Division of Epidemiology, School of Public Health, University of California, Berkeley, California, United States of America; 4 Acharya Shri Chander College of Medical Sciences and Hospital, Jammu, India; 5 Department of Epidemiology, Biostatistics and Occupational Health, McGill University, Montreal, Canada; 6 Respiratory Epidemiology and Clinical Research Unit, Montreal Chest Institute, McGill University, Montreal, Canada; 7 Division of Infectious Diseases and Medical Microbiology, McGill University Health Centre, Montreal, Canada; University College London, United Kingdom

## Abstract

**Background:**

Although interferon-gamma release assays (IGRA) are promising alternatives to the tuberculin skin test, interpretation of repeated testing results is hampered by lack of evidence on optimal cut-offs for conversions and reversions. A logical start is to determine the within-person variability of T-cell responses during serial testing.

**Methodology/Principal Findings:**

We performed a pilot study in India, to evaluate the short-term reproducibility of QuantiFERON-TB Gold In Tube assay (QFT) among 14 healthcare workers (HCWs) who underwent 4 serial QFT tests on day 0, 3, 9 and 12. QFT ELISA was repeated twice on the same sets of specimens. We assessed two types of reproducibility: 1) test-retest reproducibility (between-test variability), and 2) within-person reproducibility over time. Test-retest reproducibility: with dichotomous test results, extremely high concordance was noticed between two tests performed on the same sets of specimens: of the 56 samples, the test and re-test results agreed for all but 2 individuals (κ = 0.94). Discordance was noted in subjects who had IFN-γ values around the cut-off point, with both increases and decreases noted. With continuous IFN-γ results, re-test results tended to produce higher estimates of IFN-γ than the original test. Within-person reproducibility: when continuous IFN-γ data were analyzed, the within-person reproducibility was moderate to high. While persons with negative QFT results generally stayed negative, positive results tended to vary over time. Our data showed that increases of more than 16% in the IFN-γ levels are statistically improbable in the short-term.

**Conclusions:**

Conservatively assuming that long-term variability might be at least twice higher than short-term, we hypothesize that a QFT conversion requires two conditions to be met: 1) change from negative to positive result, **and** 2) at least 30% increase in the baseline IFN-γ response. Larger studies are needed to confirm our preliminary findings, and determine the conversion thresholds for IGRAs.

## Introduction

In many high-income countries with low rates of tuberculosis (TB), serial testing for latent TB infection (LTBI) is recommended for persons at increased risk of TB exposure, such as healthcare workers.[1] However, the conventional tuberculin skin test (TST) has known limitations in accuracy and reliability,[2] and the interpretation of repeated TST results is complicated because of non-specific variations in test results (due to test related error and biological variations over time), boosting, conversions, and reversions.[3]

Recently, *in-vitro* assays for LTBI–interferon-gamma (IFN-γ) release assays (IGRAs) have been developed. IGRAs are highly specific, especially in BCG vaccinated populations.[4] IGRAs have characteristics that are ideal for serial testing[5]: they are more specific than TST, can be repeated without concerns about sensitization and boosting, and testing protocol requires fewer visits. Unlike the TST, IGRAs do not require a baseline two-step testing protocol.

Although IGRAs have been recommended for serial testing in some countries,[6] there is still uncertainty regarding the interpretation of serial IGRA test results.[5], [7] Data are currently lacking on several key questions[5], [7]: 1) what is the reproducibility (reliability) of T cell responses over time (within-person variability over time)? 2) What is a IGRA “reversion” and what is the clinical significance of a IGRA reversion? 3) What is a IGRA “conversion” and what threshold (cut-off) should be used to define conversion? 4) How can IGRA conversions be distinguished from non-specific (random) variations in T cell responses over time? 5) What is the prognosis of a IGRA conversion and will treatment of individuals with IGRA conversions reduce their subsequent risk of progression to active TB?

Despite the licensure of two commercial IGRAs, there are limited data on the reproducibility of IGRAs, particularly with regard to within-person variability of T-cell responses during serial testing. For example, how much day to day, month to month, and year to year variability is expected with repeated IGRA testing, even in the absence of new exposure? If the same specimen is tested again, how much test-retest variation is likely? Also, there are limited data on how much IFN-γ responses will increase following new infection and how to differentiate this from changes due to test-related error or non-specific biological variations over time. In other words, how much variation in IFN-γ level is normal (i.e. expected ‘wobble’ or ‘noise’) with serial testing, beyond which increases in the IFN-γ levels likely indicates a genuine conversion (i.e. new infection)? Without such data on variations in T cell responses over time, the results of serial IGRA testing are difficult to interpret.[5]

We performed a pilot study in India, to evaluate the short-term reproducibility of QuantiFERON-TB Gold In Tube assay (QFT; Cellestis Ltd, Carnegie, Australia), a whole-blood IGRA among healthcare workers (HCWs). We assessed two types of reproducibility: 1) test-retest reproducibility (between-test variability), and 2) within-person reproducibility over time. Using these data, we attempted to derive cut-offs for QFT conversions.

## Methods

### Setting, study design and participants

Our study was done at the Mahatma Gandhi Institute of Medical Sciences (MGIMS), Sevagram, a rural teaching hospital in Central India. In previous cross-sectional[8] and longitudinal studies[9] done among the health care workers (HCWs) in this hospital, the prevalence and annual incidence of LTBI were estimated to be 41% and 5%, respectively. Both studies showed a fairly high degree of concordance between TST and QFT results.

We used a short-term longitudinal study design and a repeated measures analysis to determine the variation in the QFT results among HCWs working in high TB exposure areas of the hospital. This strategy was used as these HCWs had a high likelihood of LTBI and therefore would enable us to study the reproducibility of positive QFT results. Because HCWs in India have a high risk of TB exposure,[10] and because our prior work has demonstrated a high rate of conversions among HCWs in India,[9] long-term reproducibility was not considered feasible, given the high likelihood of new infections, and the inability to distinguish between real conversions and non-specific variations over time. Thus, we chose to study short-term reproducibility, where all repeat tests were performed within a 2 week period in March and April 2007.

After written informed consent was obtained, 14 consenting volunteers agreed to participate in the study. The study design included collection of baseline data (such as demographics, occupational category, BCG scar, and previous TB infection/disease) and collection of venous blood samples at four time-points (days 0, 3, 9 and 12). To minimize any potential circadian variation, blood for all 4 measurements was collected at the same time of the day (+/− 1 hour). Participants with at least one positive QFT result were screened for active TB disease and were referred for follow-up as necessary. The project was approved by institutional review boards (at MGIMS hospital and McGill University) and written informed consent was obtained from all volunteers.

### Testing protocol for the QuantiFERON-TB Gold In Tube assay

Venous blood was drawn into three 1 mL tubes, one containing heparin (negative control), another containing mitogen (positive control), and a third tube coated with early secreted antigen target 6 (ESAT-6), culture filtrate protein 10 (CFP-10), and TB7.7 (Rv2654) peptides. All blood samples on a particular day were collected within one hour of each other, and within two hours of collection all the tubes were incubated at 37°C for exactly 24 hours. After incubation, the harvested plasma was separated by centrifugation and stored at 2–8°C before performing ELISA. Specimens were not frozen at −20 or −80°C. Because specimens were not frozen, no centrifugation or thawing was done prior to ELISA. The blood collection procedure was video-graphed for ensuring consistency, and time logs were maintained for all of the above procedures.

The ELISA was planned in a manner that all plasma samples belonging to one individual were tested on the same 96 well microtiter plate. This was done to minimize the effect of inter-plate variation on within-person variability over time. Two ELISA runs were planned on the same set of samples–the time interval between the two assays was 1 week (during this interval, the stimulated plasma specimens were stored at 2–8°C). To minimize inter-operator and inter-laboratory variability, all assays were done by the same operator in the same laboratory. Two ELISA runs were aimed to determine if the QFT results from two different microtiter plates of the same specimens were reproducible, and if the laboratory procedures in the two runs were robust. All procedures during the ELISA runs were in accordance with the manufacturer's instructions. All assays met the quality control standards and were deemed valid (there were no indeterminate results). The cut-off value for a positive QFT test was IFN-γ≥0.35 IU/mL, as per the manufacturer's recommended threshold. According to the manufacturer, the QFT ELISA cannot accurately estimate IFN-γ levels when they exceed 15 IU/mL. Thus, values greater than 15 IU/mL should be truncated as 15 IU/mL. However, because such truncation might artificially increase the reliability of strongly positive test results, and because only 1 subject had IFN-γ>15 IU/mL, we performed data analyses without truncation.

Because test reproducibility is often influenced by the expertise of the research team, we performed this study in an institution that had considerable experience in IGRA research, as evidenced by several prior studies,[8], [9], [11], [12], [13] and a laboratory that had substantial expertise in serology and immune-based testing for infectious diseases.

### Statistical analysis

The analysis focused on two types of reproducibility: 1) test-retest (between-test) variations in IGRA results (same stimulated samples tested in two separate ELISA assays), and 2) short-term, within-person reproducibility in IGRA results over time (same person tested repeatedly, on separate occasions, over a period of time).

To examine both test-retest reproducibility and within-person reproducibility, we considered both the dichotomized test results (positive/negative, based on the ≥0.35 IU/mL cut-off) and the continuous measure of interferon-γ levels from the QFT assay (reported as IFN-γ in international units (IU) per mL).

For dichotomous measures, the number of discordant test results (both between tests and within individuals) was too few to allow for formal statistical tests. The Cohen κ statistic for concordance (agreement) was used.

For continuous measures, conventional approaches such as test-retest correlation were not used as correlation measures only linear association, not agreement of results. Therefore, the between-test reproducibility of the QFT results from the same sample of blood was assessed using a linear mixed effects analysis of the difference in test and re-test results allowing for the within-person clustering by the inclusion of random intercepts and an autoregressive correlation structure.[14], [15] For this analysis, the test and re-test results were examined on the log-scale so as to normalize their difference.

To assess the within-person test reproducibility of the continuous QFT results, only data from the initial testing of the blood samples was considered since in routine clinical or public health practice blood will not usually be stored and re-tested at a later date. A linear mixed effects model analysis[14], [15] of the log-QFT results allowing for correlation of measurements taken on the same individuals by the inclusion of random intercepts and an autoregressive correlation structure was performed. So as to be able to perform analyses on approximately normally distributed data, the response was taken to be the change in log-QFT results from the first day of testing.

## Results

### Description of study participants

Of the 14 volunteers who participated (age range 25 to 49; 4 women and 10 men), 10 were employed in the Internal Medicine department and 4 worked in laboratory services. Ten of the 14 HCWs were residents or clinical fellows. None of the participants had any immunosuppressive illness, and none reported any illness during the study period. All HCWs had BCG scars, and one HCW had been treated for TB disease in the past. TST was not performed on these volunteers, because of the concern that PPD might boost subsequent T-cell IFN-γ responses. Of the 14 HCWs, only 3 had had prior TSTs and all had negative results. Each of the 14 HCWs underwent four serial QFT assays, and therefore a total of 56 QFT test (14×4 = 56) results were available for within-person reproducibility analysis. The QFT ELISA was run twice on the same stimulated samples and therefore data on 56 test/re-test pairs were available to assess test-retest reproducibility.

### Test-retest reproducibility

Table 1 shows the initial QFT results of all 14 HCWs for each of the 4 time points. Table 2 shows the results of the repeat test performed on the same specimens after 1 week. In examining the test-retest reproducibility of dichotomous test results (i.e. test positive or negative), extremely high concordance was noticed between two tests performed *on the same sets of plasma specimens*: of the 56 blood samples drawn from 14 individuals, the test and re-test results agreed for all but 2 individuals (Table 3). Table 4 shows the IFN-γ responses for the two subjects with discordant test-retest results. Overall, 96.4% of blood samples had concordant test results, corresponding to a κ statistic of 0.94. However, these summaries do not account for the fact that 14 individual each contributed four samples, so that the 56 test/re-test pairs are not entirely independent.

**Table 1:** Within-person variability in initial QFT results (14 individuals tested 4 times on day 0, 3, 9 and 12)

ID#	Age	Sex	QFT Test 1 (Day 0)		QFT Test 2 (Day 3)		QFT Test 3 (Day 9)		QFT Test 4 (Day 12)		Overall trend across 4 tests
			IFN-γ (IU/mL)	Result*	IFN-γ (IU/mL)	Result*	IFN-γ (IU/mL)	Result*	IFN-γ (IU/mL)	Result*	
1	49	M	0.60	POSITIVE	1.44	POSITIVE	0.59	POSITIVE	0.30	NEGATIVE	Discordant
2	33	M	0.67	POSITIVE	0.77	POSITIVE	0.93	POSITIVE	0.78	POSITIVE	Positive
3	26	M	2.37	POSITIVE	5.52	POSITIVE	4.17	POSITIVE	2.41	POSITIVE	Positive
4	28	F	12.86	POSITIVE	8.44	POSITIVE	7.43	POSITIVE	5.52	POSITIVE	Positive
5	27	M	1.04	POSITIVE	1.05	POSITIVE	1.09	POSITIVE	1.44	POSITIVE	Positive
6	33	M	2.12	POSITIVE	3.48	POSITIVE	1.57	POSITIVE	0.94	POSITIVE	Positive
7	27	F	0.01	NEGATIVE	0	NEGATIVE	0	NEGATIVE	0	NEGATIVE	Negative
8	25	F	0	NEGATIVE	0	NEGATIVE	0.05	NEGATIVE	0	NEGATIVE	Negative
9	28	M	0	NEGATIVE	0	NEGATIVE	0.02	NEGATIVE	0	NEGATIVE	Negative
10	25	M	1.05	POSITIVE	0.46	POSITIVE	0.57	POSITIVE	0.57	POSITIVE	Positive
11	26	M	25.23	POSITIVE	25.23	POSITIVE	20.23	POSITIVE	16.82	POSITIVE	Positive
12	27	F	0.45	POSITIVE	0	NEGATIVE	0.02	NEGATIVE	0.02	NEGATIVE	Discordant
13	29	M	10.22	POSITIVE	6.97	POSITIVE	10.15	POSITIVE	13.62	POSITIVE	Positive
14	27	M	0.03	NEGATIVE	0	NEGATIVE	0	NEGATIVE	0	NEGATIVE	Negative

QFT: QuantiFERON-TB Gold In Tube®; IFN-γ: interferon-gamma

* The cut-off value for a positive QFT test was IFN-γ≥0.35 IU/mL

**Table 2:** Within-person variability in serially repeated (re-test) QFT results (14 individuals tested 4 times on day 0, 3, 9 and 12)

ID#	Age	Sex	QFT Test 1 (Day 0)		QFT Test 2 (Day 3)		QFT Test 3 (Day 9)		QFT Test 4 (Day 12)		Overall trend across 4 tests
			IFN-γ (IU/mL)	Result*	IFN-γ (IU/mL)	Result*	IFN-γ (IU/mL)	Result*	IFN-γ (IU/mL)	Result*	
1	49	M	0.71	POSITIVE	2.27	POSITIVE	1.07	POSITIVE	0.49	POSITIVE	Positive
2	33	M	1.1	POSITIVE	1.26	POSITIVE	1.78	POSITIVE	1.48	POSITIVE	Positive
3	26	M	3.94	POSITIVE	10.19	POSITIVE	7.34	POSITIVE	4.1	POSITIVE	Positive
4	28	F	23.35	POSITIVE	14.78	POSITIVE	11.04	POSITIVE	8.74	POSITIVE	Positive
5	27	M	1.61	POSITIVE	1.92	POSITIVE	1.87	POSITIVE	2.54	POSITIVE	Positive
6	33	M	3.12	POSITIVE	5.44	POSITIVE	2.39	POSITIVE	1.5	POSITIVE	Positive
7	27	F	0.01	NEGATIVE	0.04	NEGATIVE	0	NEGATIVE	0	NEGATIVE	Negative
8	25	F	0	NEGATIVE	0.01	NEGATIVE	0	NEGATIVE	0	NEGATIVE	Negative
9	28	M	0.01	NEGATIVE	0	NEGATIVE	0.01	NEGATIVE	0	NEGATIVE	Negative
10	25	M	0.89	POSITIVE	0.39	POSITIVE	0.45	POSITIVE	0.45	POSITIVE	Positive
11	26	M	23.35	POSITIVE	23.38	POSITIVE	17.92	POSITIVE	14.45	POSITIVE	Positive
12	27	F	0.19	NEGATIVE	0	NEGATIVE	0.01	NEGATIVE	0.01	NEGATIVE	Negative
13	29	M	9.49	POSITIVE	6.41	POSITIVE	8.83	POSITIVE	11.13	POSITIVE	Positive
14	27	M	0.18	NEGATIVE	0	NEGATIVE	0	NEGATIVE	0	NEGATIVE	Negative

QFT: QuantiFERON-TB Gold In Tube®; IFN-γ: interferon-gamma

* The cut-off value for a positive QFT test was IFN-γ≥0.35 IU/mL

**Table 3:** Test-retest concordance of two QFT tests performed on the same set of specimens (N = 56 paired specimens from 14 subjects)

		Re-test QFT result	
		Positive*	Negative
First QFT assay result	Positive*	35	1
	Negative	1	19

Concordance = 96.4%; κ = 0.94

* The cut-off value for a positive QFT test was IFN-γ≥0.35 IU/mL

QFT: QuantiFERON-TB Gold In Tube®; IFN-γ: interferon-gamma.

**Table 4:** Raw data for the two individuals with discordant QFT results; both individuals had discordant between-test results and discordant within-person results*

	Individual A (ID# 1)		Individual B (ID# 12)	
	IFN-γ (IU/mL)		IFN-γ (IU/mL)	
Test # (day)	Test	Re-test	Test	Re-test
1 (day 0)	0.60	0.71	0.45	0.19
2 (day 3)	1.44	2.27	0.00	0.00
3 (day 9)	0.59	1.07	0.02	0.01
4 (day 12)	0.30	0.49	0.02	0.01

* Complete QFT results for both individuals are shown in Tables 1 and 2

QFT: QuantiFERON-TB Gold In Tube®; IFN-γ: interferon-gamma

**Figure 1:**
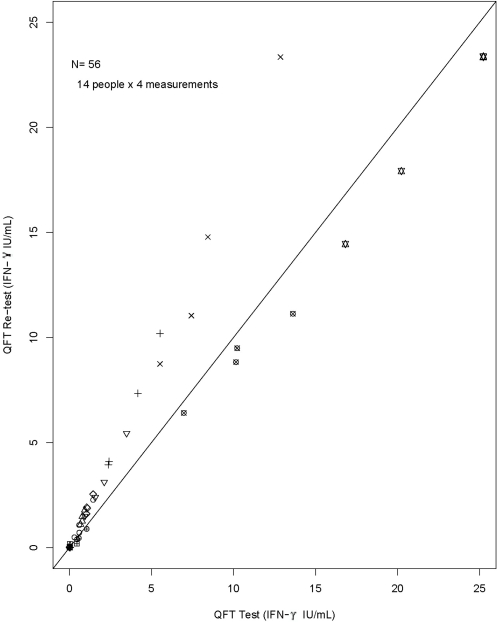
Test versus re-test QFT results expressed as IFN-γ (IU/mL). Data for each individual is plotted with different symbols, and the line of equality is shown as the diagonal. Note that more points fall above the line of equality than below, indicating higher IFN-γ levels upon re-testing of blood. The spread of the IFN-γ results from the line of equality increases with increasing IFN-γ results, indicating that a log-transformation was appropriate. QFT: QuantiFERON-TB Gold In Tube®; IFN-γ: interferon-gamma.

In examining the test-retest reproducibility of continuous IFN-γ results, re-test results tended to produce higher estimates of IFN-γ than the original test (Figure 1). As with many biological measures, the between-test variability increases with the level of IFN-γ in the sample, and so log-transformation was required to normalize the difference in IFN-γ (test–re-test) values so that appropriate limits of agreement [16], [17] could be plotted.

**Figure 2:**
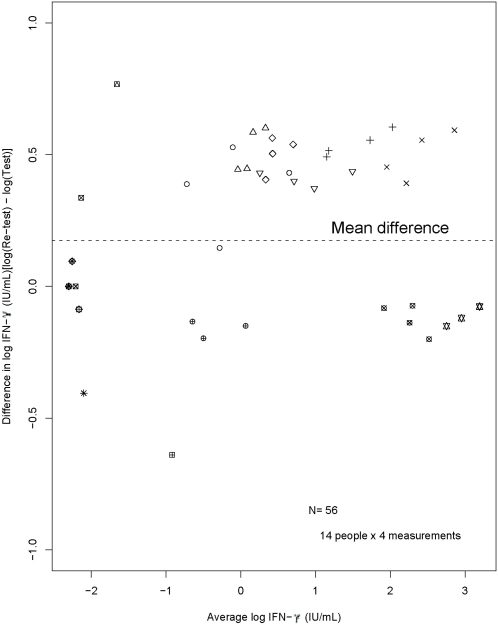
Difference in QFT results (test–re-test) versus the average of QFT test and re-test results (all on the log scale). Separate plotting characters are used for each individual and the mean log-difference is shown as the broken horizontal line. We note that there tend to be more points above zero and hence the average difference (test–re-test) lies above 0, indicating higher IFN-γ results upon re-testing of the same specimens. QFT: QuantiFERON-TB Gold In Tube®; IFN-γ: interferon-gamma.

Agreement between the two tests was moderate: the mean difference is 0.173 on the log scale, with limits of agreement of 0.021, 0.325 across the sample (Figure 2). Transforming back to the original scale, this suggests that about 95% of the time, a second test performed on the same blood sample will, on average, be 2–39% higher than the first test and will typically be 19% greater than the initial test. For example, a blood sample that yields a test result of 0.25 IU/mL, upon re-testing, is likely to yield a second result in the range of 0.26 to 0.35 IU/mL while a blood sample that yields a test result of 0.30 IU/mL if re-tested may yield a result between 0.31 and 0.42 IU/mL. Therefore, test-retest variability was high enough to suggest that concordant test results were likely when initial QFT values were less than 0.25 or greater than the positive-test threshold of 0.35 IU/mL. On the other hand, when initial test values were close to the cut-off point, repeat testing was likely to be discordant because of test-retest variability.

### Short-term within-person reproducibility

The initial testing of the samples yielded 12 individuals with concordant test results (all positive or all negative) and two individuals with three of the four tests concordant (Table 1). That is, 96.4% of individuals had concordant *initial* test results, corresponding to a κ statistic of 0.91. As shown in Table 2, the repeat tests of the same samples were concordant at each of the four time points for all individuals, so that 100% of individuals had concordant results at all four days for the blood samples that were re-tested (perfect agreement across all four days of testing).

**Figure 3:**
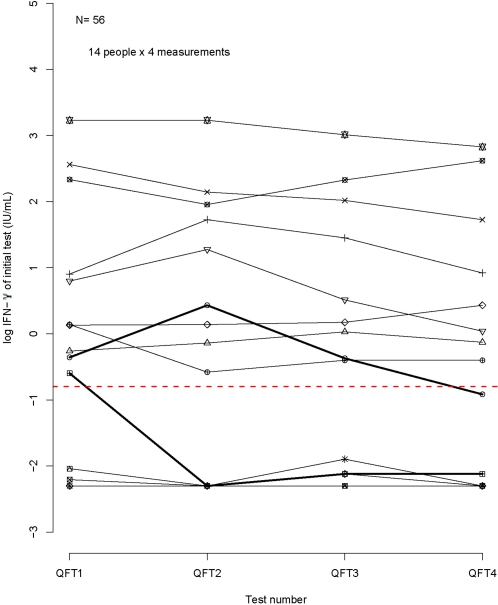
Within person variability of log IFN-γ responses over 4 time points (day 0, 3, 9 and 12). Separate plotting characters are used for each individual (N = 14) who underwent 4 QFT assays on days 0, 3, 9, and 12; the dashed red line indicates the cut-off for QFT test positivity. For each individual, the IFN-γ results taken on four days are plotted on the log scale. Most individuals do not cross the positive test result threshold. For two individuals (lines shown in bold) whose trajectories do cross the threshold, test results are discordant. QFT: QuantiFERON-TB Gold In Tube®; IFN-γ: interferon-gamma.

Over the course of the study, continuous log IFN-γ results from the initial test tended to decrease (Figure 3). The mixed model analysis confirmed this, finding a slight but significant decrease of 26% (1.1 to 44.8%) in the QFT results on day 12 as compared to day 0 (p = 0.049). QFT results on days 3 and 9 did not differ significantly from the first day of testing. This indicates that the test was reliable over the short-term, but repeatability of the actual continuous test values (rather than the dichotomous results) might slightly decrease over an interval of two weeks or more.

Because of the potential for drift in QFT results over the “medium term,” a secondary analysis was undertaken on the *change* in log-QFT results between successive visits. We fit a linear mixed effects model with random intercepts only, as this facilitates estimation of the intra-class correlation (ICC) statistic, calculated as the ratio of the within-person variability to the total variability. We first considered a model that adjusts for testing day, and no longer observed a significant effect of day. We therefore proceeded to fit a model without covariates which accounts for the within-person correlation through the random intercepts. This model yielded an estimate of the ICC of 0.92. Based on this model, we found no significant change in QFT results over time: estimated change between visits was a decrease of 30% (95% CI: −57.3% to +16.0%). This suggests that an increase in QFT of more than 16% in a short period indicates a change that is unlikely to have occurred through random fluctuations alone and may in fact be attributable to a genuine change in TB infection status over a short time period.

## Discussion

IGRAs are promising alternatives to the tuberculin skin test, and their use is rapidly expanding.[4] However, little evidence is available to guide the interpretation of repeated (serial) testing results.[5], [7] Existing studies suggest that conversions, reversions and non-specific variations occur with IGRA serial testing, just as they do with TST serial testing.[9], [18], [19], [20], [21] Serial testing studies suggest that IGRAs are highly dynamic tests and T-cell responses, especially weakly positive responses, tend to fluctuate over time, even in the absence of specific treatment.[9], [18], [19], [20] There is growing evidence that IGRAs may be inherently prone to conversions and reversions, and this dynamic characteristic raises concerns as to whether these assays are too labile or lack in reproducibility for serial testing.[5] Although IGRAs are often thought of as tests that produce dichotomous (yes/no) results, studies show that these tests are threshold dependent (i.e. depend on the cut-off points used), and that the optimal thresholds to distinguish new infections from non-specific variation are yet to be defined.[9], [18] Also, the need for reproducibility studies has been articulated in several recent reviews.[4], [5], [7]

The results of our pilot study of short-term reproducibility suggest the following: When QFT results are interpreted using a dichotomous (positive/negative) approach, then test-retest and within-person reproducibility is very high. This may be due, in part, to the fact that most of the individuals in the study had QFT results that were well below or well above the cut-off of IFN-γ 0.35 IU/mL. Negative QFT results, for example, were almost always zero or very close to zero. Discordance was mostly noted in subjects who had IFN-γ values around the cut-off point. This is expected and is in line with previous studies that have shown a high frequency of reversions among individuals with weakly positive IGRA results (i.e. IFN-γ responses close to the test cut-off for positivity) and among individuals with an initial discordant result with TST.[5], [9], [18], [19], [20], [22] Therefore, health professionals who interpret serial QFT results should interpret weakly positive IGRA results with caution, especially if the weakly positive IGRA result is discordant with the tuberculin skin test.

When QFT results were interpreted using the continuous IFN-γ response data, the reproducibility was moderate to high. While those with negative QFT results (i.e. values close to 0 IU/mL) generally stay negative, positive results tend to vary over time. While variation in strongly positive IFN-γ values is unlikely to have any clinical impact, small variations in weakly positive IFN-γ values will most likely cause discordance during serial testing. Overall, the impact of variations around the cut-point would depend on how frequently IFN-γ values are found close to the cut-off point. In a previous cross-sectional study of 726 HCWs in India,[8] we found that only about 5% of the cohort has IFN-γ values between 0.25 and 0.45 IU/mL. This suggests that borderline values around the cut-point are probably uncommon (although this would need to be verified in all settings), and therefore minor variations due to QFT reproducibility may not have a serious impact on serial testing results.

One of our study aims was to generate preliminary data on how much variation in IFN-γ level is normal with serial testing and how much increase in the IFN-γ level might indicate a genuine conversion. This is an important issue for the identification and treatment of IGRA conversions. To identify IGRA conversions, there must be clarity on how to define a conversion. A simplistic definition of QFT conversion, proposed by the US Centers for Disease Control and Prevention (CDC), is a “change from a negative to a positive result.”[1] This definition is easy to use from an operational perspective, but does a relatively small increase in IFN-γ responses constitute a true conversion? What if, for example, the IFN-γ level increased from 0.34 to 0.35 IU/mL? Is that a real conversion that warrants 9 months of isoniazid preventive therapy? A TST increase from 9 mm to 10 mm will usually not be considered a true conversion,[23] because it is known that biologic and test-related variations result in changes of <6 mm induration in 95% of subjects.[3] Therefore, TST increases of ≥6 mm are considered to represent a true biologic phenomenon.[3] Applying the same line of reasoning, a minor increase from 0.34 to 0.35 IU/mL may not be a conversion; it may merely reflect non-specific IFN-γ variability over time.

Our linear mixed effects model results showed that increases of more than 16% in the IFN-γ levels are statistically improbable. Thus an increase beyond 16% in the baseline IFN-γ level may indicate new infection (i.e. conversion). However, this estimate of 16% reflects only short-term reliability. Conservatively assuming that long-term within-person variability might be higher than what we found (especially since inter-plate and inter-observer variation is also likely in routine practice), one approach would be to inflate the expected variation to 30%, about twice what we found. With this assumption, it may be reasonable to hypothesize that a QFT conversion requires two conditions to be met: 1) change from negative to positive result, **and** 2) at least 30% increase in the baseline IFN-γ response.

For example, if the baseline IFN-γ was 0 IU/mL, and the repeat IFN-γ was 0.35 IU/mL, this would meet both conditions for conversion. However, if, for example, the baseline IFN-γ was 0.30 IU/mL, and the repeat IFN-γ was 0.35 IU/mL, this would meet the first condition but not the second, because the IFN-γ did not increase by 30%. Future studies must validate our proposed criterion for QFT conversion, and establish the prognosis of IGRA conversions, especially conversions that are ‘weak’ (i.e. associated with minor increases around the cut-off value) versus conversions that are ‘strong’ (i.e. associated with large increases in IFN-γ levels). Until then, health professionals should be cautious about using a simplistic negative to positive definition of conversion, and instead consider the amount of change in absolute IFN-γ responses, as well as relevant clinical information to detect and treat conversions. To facilitate this, laboratories must report dichotomous as well as continuous IGRA results.

Our study had limitations. As a pilot study, it provided useful preliminary data, but the results are not definitive. Test reproducibility determined in a research setting may not reflect reproducibility in field settings. Because our study was done in a TB endemic setting with potential for ongoing exposure, the results may not necessarily reflect the reproducibility of IGRAs in settings with low incidence of TB. In addition, our study does not provide any data on long-term reproducibility of IGRA results. Since most serial testing programs recommend annual TB screening,[1] long-term IGRA reproducibility needs to be determined in future studies. Ideally, such studies must be done in settings where ongoing TB exposure is unlikely (to avoid confusing real conversions with non-specific changes in T-cell responses). Such studies must generate data on inter-plate, inter-observer and inter-laboratory variations in IGRA results. It is also necessary to evaluate if fresh samples produce different results than frozen samples (because of the potential problem of evaporation of plasma water during cold storage and thawing). Also, our study results cannot be extrapolated to all IGRAs. ELISA and ELISPOT-based assays may have differing reproducibility characteristics.

Serial testing studies are ongoing in countries such as Canada and the US, and should provide definitive evidence on serial IGRA testing and reproducibility. Ongoing cohort studies, described elsewhere,[24] should also provide useful data on the prognosis of IGRA conversions. Lastly, because our study showed variations in test-retest results, there is a need to explore the use of automated ‘robotic’ systems (that perform all the ELISA steps including pipetting of reagents, plate shaking, etc.) in reducing such variations, and compare the performance and costs of automated versus manual ELISA.
